# Prognostic factors for disability claim duration due to musculoskeletal symptoms among self-employed persons

**DOI:** 10.1186/1471-2458-11-945

**Published:** 2011-12-22

**Authors:** JM Richter, BM Blatter, J Heinrich, EMM de Vroome, JR Anema

**Affiliations:** 1 Netherlands Organisation for Applied Scientific Research TNO, Hoofddorp, the Netherlands; 2 Body@Work, Research Center on physical activity, work and health, TNO-VU/VUmc, Hoofddorp, the Netherlands; 3 Department of Social Medicine, EMGO-institute for health and care research, VUmc, Amsterdam, the Netherlands; 4 Research Center for Insurance Medicine AMC-UWV-VUmc, Amsterdam, the Netherlands

**Keywords:** Work disability predictors, special worker populations, musculoskeletal problems

## Abstract

**Background:**

Employees and self-employed persons have, among others, different personal characteristics and different working conditions, which may influence the prognosis of sick leave and the duration of a disability claim. The purpose of the current study is to identify prognostic factors for the duration of a disability claim due to non-specific musculoskeletal disorders (MSD) among self-employed persons in the Netherlands.

**Methods:**

The study population consisted of 276 self-employed persons, who all had a disability claim episode due to MSD with at least 75% work disability. The study was a cohort study with a follow-up period of 12 months. At baseline, participants filled in a questionnaire with possible individual, work-related and disease-related prognostic factors.

**Results:**

The following prognostic factors significantly increased claim duration: age > 40 years (Hazard Ratio 0.54), no similar symptoms in the past (HR 0.46), having long-lasting symptoms of more than six months (HR 0.60), self-predicted return to work within more than one month or never (HR 0.24) and job dissatisfaction (HR 0.54).

**Conclusions:**

The prognostic factors we found indicate that for self-employed persons, the duration of a disability claim not only depends on the (history of) impairment of the insured, but also on age, self-predicted return to work and job satisfaction.

## Background

Although many high quality studies have focused on risk factors for the development of musculoskeletal symptoms [1-3], relatively little is known about prognostic factors for the duration of work disability due to musculoskeletal symptoms [4]. This is remarkable, since musculoskeletal pain - and in particular pain in the neck/shoulder and low back regions - has been shown to be strongly associated with long-term sickness absence [5]. Thus, in order to prevent health care costs and personal suffering, information about this topic is of great importance for both the development of specific interventions directed at these prognostic factors as for the identification of people at risk for a long-term work disability period. Although long-term absences only constitute a small fraction of all absence periods, they comprise up to 75% of all absence costs [6,7].

In recent years a few studies on prognostic factors for low back pain (LBP) have been published [5,8-14]. One important message which emerges after reviewing these studies is that more high quality prognostic studies for return to work (RTW) after an episode of back pain are needed in which multiple factors are measured and analyzed simultaneously. Comparable studies on other musculoskeletal symptoms, like neck and upper extremity pain are also still limited although neck pain is the second most prevalent musculoskeletal symptom [15]. A review of Dekkers-Sanchez et al. [16] paid attention to prognostic factors associated with long-term sick leave for several different disorders, while a review of Mallen et al. [17] was restricted to prognosis of musculoskeletal pain without special attention to sick leave. Recently, two studies have focused on both duration of sick leave and musculoskeletal symptoms on several locations [4,5].

All the above mentioned studies are targeting employees, while information on self-employed persons is lacking [18]. In the Netherlands, 12.5% of the Dutch labor force consists of self-employed persons and their number is growing [19]. It is generally known that there are differences between employees and self-employed persons which may influence both the onset of sick leave and the prognosis of claim duration [20]. For instance, self-employed persons are characterized by high levels of intrinsic motivation to work (long working hours), job control, job insecurity, work demands, decision latitude, type-A personality, and low levels of social support in their work [21]. Furthermore, self-employed persons have little guidance in returning to work since they do not have access to an occupational physician. Finally, also factors related to differences in the compensation system in case of illness create the need to distinguish between employees and self-employed persons.

Studies on determinants to predict long-term sickness absence in self-employed persons are extremely scarce [18]. In the Netherlands, only a few relevant studies focus on sick leave in self-employed persons, but most studies focus on specific occupations instead of the general population of self-employed persons [13,22]. Moreover, all studies deal with a broad range of sick leave aspects without a focus on prognostic factors [13,22,23]. Finally, in a study published previously [24] we found no effects of an intervention including physical training with a cognitive behavioral component. Because of all these factors, we were interested to study in self-employed persons which factors are determinants of longer claim duration. We used the longitudinal data of the above mentioned RCT and added to the longitudinal data subjects who filled in questionnaires but refused to participate in the intervention study.

Therefore, the purpose of the present study is to identify prognostic factors for the duration of a disability claim due to musculoskeletal symptoms among all kinds of self-employed persons in the Netherlands.

## Methods

The study was a cohort study with a follow-up period of one year, in which possible prognostic variables at baseline were associated with the duration of the disability claim in multivariate analyses.

### Study population

The study population consisted of self-employed persons insured by a large Dutch insurance company that provides work disability insurances. The source population (n = 54.000) consisted of self-employed persons in all parts of the Netherlands. In the Netherlands - as in many other European countries - income insurance for the self-employed is optional as of 2004, resulting in only 50% of the self-employed persons in the Netherlands being income insured [24]. All persons with a new claim episode from November 2004 until December 2006 were invited to take part in the study if they met the following inclusion criteria: (1) having non-specific musculoskeletal symptoms and (2) being unable to fulfill their job for more than 25% according to a medical assessment.

**Figure 1:**
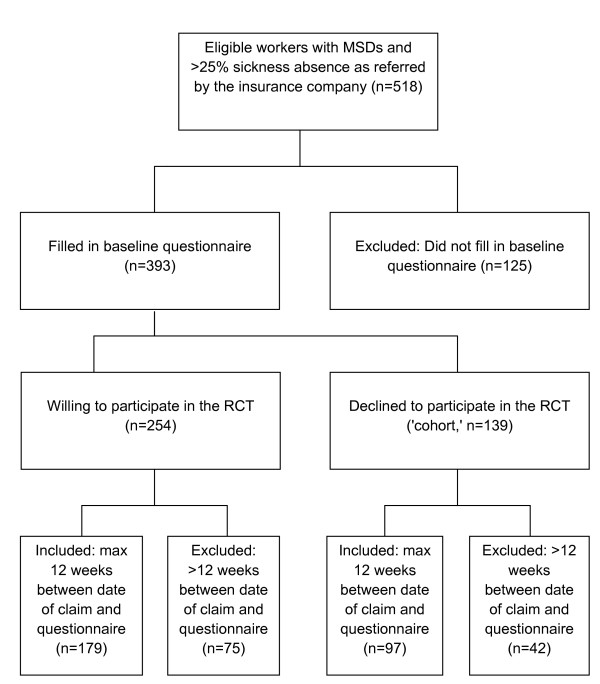
**Flow diagram describing the inclusion process of participants for the analyses**.

During the study inclusion period, 518 self-employed persons with a work disability claim were referred to the research assistant. Data of a total of 393 self-employed persons were included in the present study. The majority of these data (65% or 254 persons) came from self-employed with MSDs who were willing to participate in an RCT (Randomized Controlled Trial). One third (139 or 35%) were participants who declined to participate in the RCT but were willing to fill in the questionnaire and therefore were classified as the 'cohort' group. The cohort group received usual care. Both the RCT and cohort group filled in a baseline questionnaire on individual, disease- and work-specific questions. The RCT was set up to examine the effectiveness of physical training with and without a cognitive behavioral component and workplace specific exercises (for more information on the RCT, see Heinrich et al. [24]). The main reasons why the cohort group refused to participate in the RCT were reluctance to be randomized and reluctance to do physical training. In order to minimize bias, the characteristics of the RCT group and the cohort group were first compared for differences in the other prognostic factors. Since no relevant differences were found between the RCT and the cohort, and since we only used the baseline questionnaire to identify prognostic factors before the actual treatment was started, we decided not to include willingness to participate as a possible prognostic factor, but to adjust the analyses for this factor. When someone met the inclusion criteria he or she received written and oral information about the study purpose and procedures and was enrolled after giving informed consent. The Medical Ethics Committee of the University Medical Center in Leiden, the Netherlands, approved the study design, protocols, procedures and informed consent procedure. We decided to include only persons with a maximum of 12 weeks between the onset of their work disability claim and filling in the baseline questionnaire, in order to minimize bias caused by a mixed population that is both acute and chronic [10]. Due to this selection, we excluded 30% of the original subject group, leaving a total of 276 persons for the analyses. We clarified this process with a flow diagram, which is depicted in Figure 1.

### Potential prognostic factors

The selection of relevant prognostic factors was partly performed by reviewing recent literature on prognosis for chronic musculoskeletal symptoms [4,8-13,16,17,22]. Besides prognostic factors known from the literature, we developed a list of additional prognostic factors for sickness absence that might be specific for self-employed, i.e. financial factors and insurance-related factors, which are specified below. Based on the literature review and the additional prognostic factors we composed a list of potentially relevant prognostic factors. Based on the International Classification of Functioning, Disability and Health (WHO, 2001) we distinguished the factors in the following four ICF constructs 1) personal factors, 2) physical body functions, 3) environmental factors and 4) mental body functions. Special attention was paid to potential prognostic factors related to the insurance system. The potential prognostic factors in our study were assessed by means of the baseline questionnaire. Additional information was gathered by the electronic database of the insurance company.

Personal factors were gender, age, general health and marital status. General health was assessed by one question with five answering categories (for the analyses, these were reduced to two categories, indicating good or bad health).

Concerning physical body functions, information was collected on the history of similar musculoskeletal symptoms (yes/no), the location of musculoskeletal symptoms (categorized into 'upper extremity symptoms', 'back symptoms', 'lower extremity symptoms' and 'symptoms at multiple locations'), the duration of symptoms prior to the baseline questionnaire (weeks) and treatment by general practitioner or (para-)medical specialist at baseline (yes/no). The level of perceived pain in the previous 6 months was measured by one question on an 11-point numerical scale ranging from 0 (no pain) to 10 (very severe pain) [25]. Functional disability of the participants was assessed by the Neck Pain Disability Index (NDI, range 0-50, recalculated to 0-100%; a higher score indicating a higher disability) and the Quebec Back Pain Disability Scale (QBPDS, range 0-100) [26,27]. The scores of the NDI and QBPDS were divided into tertiles (cut-off values for the first and second tertile were 29 and 43 for NDI and 28 and 42 for QBPDS).

With regard to potential environmental prognostic factors, we included financial and insurance-related factors. The financial factors we included were hiring extra work capacity since the claim was filed (yes/no) and the perceived financial situation of the company ('(very) bad/rather give no answer' versus '(reasonably) good/(very) good'). Subscales of questionnaires were not calculated if more than 20% of the questions had missing values. Insurance-related factors were the deferment period and the level of benefit compensation. The deferment period is the time between the onset of sick leave and the start of the sickness compensation payment by the insurer. This is a period which usually varies between a few weeks and a few months, and is meant to keep costs acceptable and to prevent someone from using compensation payment for relatively minor health problems. The level of benefit compensation is chosen by the insured person.

As for the construct 'mental body functions', the Dutch version of the Tampa Scale for Kinesiophobia (TSK-DV) was used to measure fear of movement/(re)injury [28]. The score (range 0-100) was dichotomized according to previous research [29]; high kinesiophobia was classified as having a score higher than 37, low responders smaller than or equal to 37. For self-predicted timing on return to work (RTW), persons had to answer the question "When do you think you will be able to work fulltime again?" The answering categories were combined into 'within one month', 'more than one month/never' and 'no idea'. Information was gathered about job satisfaction (with categories '(very) satisfied' and '(very) unsatisfied'). Furthermore, the perceived intensity of physical work was measured by means of the Dutch Musculoskeletal Questionnaire (DMQ), for which the mean score of all factors was used in the analysis [30]. Psychological job demands (work pace and work quantity) were measured by means of a Dutch version of the Job Content Questionnaire [31], and this scale was divided into tertiles (cut-off values 36 and 45).

In the analyses, the above mentioned prognostic factors are assumed to represent a constant hazard ratio (HR) over time, meaning that a prognostic factor has the same influence on claim duration throughout the claim period. However, a recent study has shown that certain prognostic factors for return to work after sickness absence due to musculoskeletal symptoms have a significant interaction with time; perceived physical workload and functional disability [4]. Therefore, a decision was made to include three additional variables with an interaction with time to the analyses: NDI, QBPDS and perceived intensity of physical work. In total, 24 variables were analyzed as potential prognostic factors.

### Outcomes

The primary outcome variable claim duration was defined as the number of calendar days the participant received work disability compensation between completion of the baseline questionnaire and one-year follow up, without adjustment for the level of work disability (gross duration). The end of a claim period was defined as having less than 25% work disability according to a medical assessment, with a minimum duration of 4 weeks. This means that recurrences of work loss due to the same disorder within 4 weeks of the end of the claim were considered as belonging to the same first continuous claim period. Since only a few recurrent claim periods occurred within one year, we decided to include only the first claim period from baseline in the analyses. Data on claim duration and level of work disability were continuously collected by means of the electronic records of the insurance company, which have been shown in other studies as valid and reliable measures [32].

### Statistical analysis

To determine prognostic factors for the duration of sick leave, the data were analyzed using the Cox proportional hazards model. The primary outcome of the analysis was claim duration (days) at one year follow-up. Subjects were right-censored when they did not finish their disability claim after 12 months follow-up. For analytic purposes, these subjects were assigned to have a claim duration period of 365 days. The hazard ratio (HR) was used to indicate the effect of a prognostic factor on claim duration. A HR of 1 means that the factor had no effect on duration of a claim, a HR larger than 1 means that the factor had a 'positive' effect on claim duration, i.e. those persons finished their claim earlier than the reference group. As the first step in the analyses, univariate Cox analyses were performed for all 24 potential prognostic factors. All prognostic factors that reached a significance level of ≤ 0.10 in the univariate analysis - including the factors with time interaction - were included into the multivariate model. Age and gender, however, were forced into the multivariate model, irrespective of their level of significance in the univariate analysis. All variables were entered simultaneously into the model, and were kept into the model when significant at 5%. The survival curve and survival table were calculated with the Kaplan-Meier procedure. All analyses were performed with SPSS Statistics version 17.0.

## Results

### Subject characteristics

In total, the data of 276 persons were analyzed, of which 93% were men. A description of all analyzed prognostic factors is shown in Table 1. Of all participants, 61% were working in the agricultural sector, 15% in construction, 7% in the business sector and 17% in different sectors. The mean age was 45 years (SD 7), and 61% had a history of musculoskeletal symptoms.

**Table 1:** Univariate and multivariate Cox regression analyses of the potential prognostic factors contributing to claim duration in self-employed persons

Individual Personal factors - N(%)	Categories	Total N = 276	Univariate HR^1^	*p*	Multivariate HR (95% CI)	*p*
Age	- ≤ 40 year	70 (25%)	1	-	1	-
	
	- 41-50 year	131 (48%)	0.89	0.47	0.54 (0.36-0.80)	0.002
	
	- > 50 year	74 (27%)	0.96	0.81	0.64 (0.41-0.99)	0.05

Gender	- female	20 (7%)	1	-	1	-
	
	- male	256 (93%)	1.25	0.45	1.59 (0.78-3.21)	0.20

General health	- poor health	36 (13%)	1	-	1	-
	
	- good health	240 (87%)	1.51	0.06	0.90 (0.54-1.49)	0.68

Marital status	- living alone	25 (8%)	1	-		
	
	- living together	251 (92%)	0.83	0.42	-	-

**Physical Disease-related factorsbody functions**						

History of similar symptoms -yes	- no	108 (39%)	1	-	1	-
	
	- yes	168 (61%)	1.50	0.004	2.20 (1.52-3.18)	< 0.001

Pain severity prev. 6 months	mean (sd)	5.6 (2.3)	0.94	0.02	0.96 (0.89-1.04)	0.32

Location musculoskeletal symptoms	- no symptoms	56 (21%)	1	-	1	-
	
	- upper extremity	33 (12%)	0.82	0.40	1.16 (0.64-2.10)	0.63
	
	- back	55 (20%)	0.95	0.78	1.04 (0.63-1.72)	0.88
	
	- lower extremity	23 (8%)	0.47	0.01	0.49 (0.23-1.06)	0.07
	
	- multiple locations	106 (39%)	0.58	0.003	0.72 (0.45-1.17)	0.18

Duration of symptoms	- < 2 months	107 (39%)	1	-	1	-
	
	- 2-6 months	90 (33%)	0.65	0.007	0.75 (0.50-1.14)	0.18
	
	- > 6 months	77 (28%)	0.53	< 0.001	0.60 (0.38-0.95)	0.03

Functional status neck pain (NDI)	- little pain	90 (33%)	1	-	1	-
	
	- medium pain	78 (29%)	0.76	0.11	0.72 (0.45-1.16)	0.18
	
	- much pain	103 (38%)	0.67	0.01	0.56 (0.30-1.02)	0.06

Functional status back pain (QBPDS)	- little pain	87 (34%)	1	-	1	-
	
	- medium pain	90 (34%)	0.74	0.08	1.28 (0.81-2.03)	0.29
	
	- much pain	84 (32%)	0.78	0.14	1.42 (0.75-2.69)	0.29

Interaction: NDI * time		-	1.00	0.26	-	-

Interaction: QBPDS * time		-	1.00	0.24	-	-

Treatment by GP or (para)medical specialist	- no	36 (13%)	1	-		
	
	- yes	240 (87%)	0.86	0.46	-	-

**External Environmental factors**						

Hired employee since claim--yes	- no	173 (62%)	1	-		
	
	- yes	103 (38%)	0.84	0.22	-	-

Financial situation company	- not good	31 (11%)	1	-		
	
	- good	245 (89%)	0.83	0.39	-	-

Insured daily compensation (Euro)	- 0-50	56 (21%)	1	-	1	-
	
	- 50-75	97 (36%)	1.37	0.12	1.40 (0.88-2.22)	0.16
	
	- 75-100	90 (33%)	1.52	0.03	1.52 (0.97-2.39)	0.07
	
	- > 100	26 (10%)	1.21	0.49	1.51 (0.80-2.87)	0.21

Deferment period-- > 14 days	- ≤14 days	161 (58%)	1	-	1	-
	
	- > 14 days	115 (42%)	0.70	0.01	0.83 (0.59-1.17)	0.29

**Other factorsMental body functions**						

Fear of movement	- low fear	111 (37%)	1	-	1	-
	
	- high fear	165 (63%)	0.73	0.03	0.94 (0.67-1.33)	0.74

Self-predicted timing RTW	- < 1 month	56 (21%)	1	-	1	-
	
	- > 1 month/never	82 (30%)	0.28	< 0.001	0.24 (0.15-0.38)	< 0.001
	
	- no idea	134 (49%)	0.25	< 0.001	0.23 (0.15-0.34)	< 0.001

Job satisfaction	- not satisfied	38 (13%)	1	-	1	-
	
	- satisfied	238 (87%)	1.42	0.10	1.85 (1.13-3.04)	0.02

Perceived intensity of physical work	range 1-4, higher is heavier	mean: 2.8 (sd 0.76)	0.90	0.23	-	-

Interaction: perceived physical work * time		-	1.00	0.16	-	-

Work pace/quantity (job demands)	- low	79 (29%)	1	-		
	
	- medium	98 (37%)	1.31	0.11	-	-
	
	- high	91 (34%)	1.00	0.98		

Willingness to participate in a RCT	- yes	180 (65%)	1	-	1	-
	
	- no	96 (35%)	1.59	0.001	1.41 (1.02-1.92)	0.04

^1^A HR of > 1 indicates a shorter time to RTW

### Duration of sickness absence

The median duration of a disability claim for the whole population was 140 days (95% confidence interval (CI) 116-164 days). After one year follow-up, 218 subjects (79%) had returned to work. At 3 months, 38% of the population had returned to work; at 6 months, 58%; and at 9 months, 70%.

### Cox regression analyses

Table 1 shows the univariate and multivariate Cox regression analyses to determine the prognostic factors contributing to claim duration. In the table, the reference category is indicated with a HR (hazard ratio) of 1. Of the 24 prognostic factors analyzed in the univariate Cox regression analysis, 13 factors were significantly associated with claim duration and 15 factors (including gender and age category) were entered into the multivariate model. The multivariate model had 33 cases with missing values, leaving data from 243 persons for the multivariate model. None of the variables with interactions with time reached significance; they were therefore not included in the multivariate model.

The last two columns of Table 1 represent the HR's and p-value of the multivariate analysis. The following five variables (after adjusting for willingness to participate) influenced claim duration at one year follow-up in the multivariate analyses: age, history of similar symptoms, duration of symptoms, self-predicted timing of return-to-work and job satisfaction. Age was a significant determinant at multivariate, but not as univariate analysis.

Higher age (over 40 years) was associated with longer claim duration. Having a history of similar musculoskeletal symptoms was associated with shorter claim duration than persons that never had similar symptoms before baseline. Persons having had symptoms for more than six months at baseline had longer claim duration than subjects with symptoms for less than two months at baseline. Furthermore, self-predicted timing was a good predictor of claim duration; persons that estimated their return to work to be within one month had shorter claim duration than persons that estimated their return to work in more than a month or even 'never'. Also, persons who had no idea of when they would return to work also had longer claim duration compared to persons estimating their return to work within one month. Persons who were (very) satisfied with their job had shorter claim duration than persons who were (very) dissatisfied with their job.

**Figure 2:**
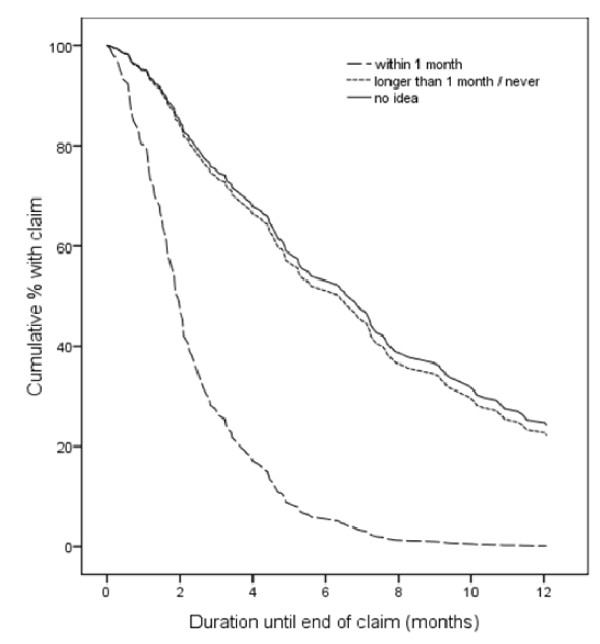
**Survival graph, stratified by self-predicted time to return to work**.

As illustration, the result of one of the six significant prognostic factors, self-predicted time to return to work, is shown in Figure 2. From this survival graph, it becomes clear that estimating time to return to work in an early phase of sickness absence is an important prognostic factor for claim duration.

## Discussion

The present study has given insight into the prognostic factors that influence the length of claim duration of self-employed persons in the Netherlands. The prognostic factors that were associated with *shorter *claim duration were younger age, having had similar symptoms in the past, having had symptoms at baseline for less than two months, self-predicted return to work within one month and job satisfaction. These factors were part of the ICF constructs 'personal factors', 'physical body functions' and 'mental body functions'. The environmental factors we studied did not significantly influence the length of claim duration.

### Comparison with findings in the literature

There has been little previous research on prognostic factors for sick leave due to musculoskeletal symptoms in self-employed persons. First of all, the primary outcome measure in the current study was claim duration, while comparable studies on employees and self-employed persons often focus on return-to-work. We pointed out earlier that the end of a claim period does not necessary equal full return to work [13]. Therefore, claim duration in the present study can only be interpreted as a proxy for time to full return-to-work. We will compare the current results with studies of self-employed and employees, and will make an attempt to find explanations for possible differences with prognostic factors in employees.

Age was found to be positively associated with the duration of sick leave, similar to the studies of van Doorn et al. and Spierdijk et al. in the self-employed [13,23]. Age was found to be a major prognostic factor for longer duration of sick leave in studies of employees as well [10]. In the current study, more than 50% of the research population consisted of farmers with concomitant high physical exposure. Previous research has found that the capacity for physical work declines with age, leading to an increase in musculoskeletal symptoms with age in physically demanding occupations [33].

The duration of current musculoskeletal symptoms was found to be positively associated with claim duration. Van Doorn analyzed the prevalence of low back disability amongst self-employed dentists, veterinarians, physicians and physical therapists, and found that sick leave duration due to low back pain was significantly associated with low back problems before insurance acceptance [13]. Similarly, Heymans et al. found a longer duration of symptoms at study inclusion to be a relevant prognostic factor for long-term sick leave due to low back pain, although this study was directed at employees [14]. The duration of symptoms can be related to the severity of symptoms, which in turn may be responsible for longer sickness absence. The factor 'duration of current complaint episode' (at baseline) indicates that treatment of musculoskeletal symptoms should start as soon as possible after the onset of sick leave or even when persons who experience MSD are still at work.

Persons who had a history of similar musculoskeletal symptoms had a shorter disability claim period than persons not having had a similar symptom episode in the past. This is an indication that having coped with similar symptoms in the past can help during recurrent periods of symptoms. No other studies in self-employed have looked at this factor, and a review by Steenstra et al. in employees did not find an effect of history of low back pain on the duration of sick leave due to low back pain [10]. Although more research in self-employed has to be done to strengthen the current results, this study indicates that having had a history of similar MSD is more important in self-employed than in employees. Even though the effects of duration of current symptoms and having a history of similar symptoms seem controversial, they can both occur. While having a history of similar complaints may help in understanding and coping with current symptoms and thus diminishing a claim duration, the duration of current symptoms is related to the severity of symptoms, thereby increasing the claim duration.

Furthermore, we found that self-predicted timing of return to work was a major prognostic factor for the duration of sickness absence. To our knowledge, no other studies on prognostic factors of sick leave duration in self-employed persons have looked at this variable, even though a recent systematic review found strong evidence that recovery expectation regarding return to work is a robust predictor of work outcome in employees with non-specific low back pain [9]. So, asking someone about their expected recovery in an early stage of sickness absence has shown to be important for both employees and self-employed persons. Apart from that, this factor may act as an invitation to practitioners to investigate further which factors may cause a person to have low expectations about returning to work [9].

Similar to our findings in the present study, moderate to low job satisfaction was found to increase claim duration in two studies on employees [8,34]. On the other hand, a recent review from Iles et al. found strong evidence that job satisfaction is *not *a predictor for work outcome [9]. No studies were found in the self-employed that looked at job satisfaction. Although conflicting results were found in studies on employees, the current study indicates that job satisfaction is an important factor in claim duration. Given the fact that self-employed persons have a higher intrinsic motivation to work than employees [21], this indicates that job dissatisfaction may influence disability claim duration to work to a much larger extent than it would in employees.

Although we expected environmental factors (financial and insurance-related) to have prognostic value for the duration of a disability claim, none of the environmental factors contributed significantly to the final model, although the insured daily compensation was borderline significant (p = 0.07). Two of the environmental factors (hired employee since claim, and perceived financial situation of the company) were not known from previous studies. A possible explanation for the fact that these two factors did not reach significance might be the wording of the question. Whether or not people hired an additional employee since the disability claim, might not necessarily be the (sole) cause of their disability. Furthermore, the answers on the financial situation of the company might be biased by social desirability.

### Considerations/Study strengths and limitations

Some limitations and strengths of the study must be considered for a solid interpretation of the results. Firstly, since we only used the baseline questionnaire in the analyses, and the time to return to work was monitored by the insurance company, loss to follow-up was not applicable in the current study.

Secondly, the factor 'willingness to participate in a RCT' was only adjusted for in the univariate and multivariate analyses and not used as a prognostic factor. The main barriers to participate in a RCT in this study were reluctance to be randomized and reluctance to do physical training. These are barriers commonly found when recruiting RCTs [35]. When this 'cohort' group is not included in the analyses, as commonly occurs, the representativeness of the study population is diminished. By analyzing both groups in the current study, this bias was minimized. Although claim duration was shorter in the group which did not participate in the RCT, separate analyses showed that including both groups in the analyses did not lead to bias in the prognostic factors that were found.

Thirdly, in the current study, 61% of the study population consisted of agricultural workers. Although few recent numbers are available on professions of self-employed persons, in the Netherlands, about 10% of all self-employed persons worked in the agricultural sector in 2009 (Statline, Central Bureau for Statistics). Our population of self-employed therefore overrepresents the agricultural sector. A previous study with a partly overlapping research group performed a sick leave analysis among self-employed Dutch farmers [22]. They estimated that the nature of work for self-employed farmers has changed in the past two decades from primarily physically demanding towards more mentally demanding tasks. This will result in job tasks that are more similar to job tasks of other self-employed persons. However, despite of the shift in tasks, some other characteristics of farmers may still differ from those in other self-employed occupations. The results of this study should therefore be interpreted with the overrepresentation in mind. Moreover, the differences we found between self-employed and employed persons may be caused by the use of different study methods in the literature for the two groups.

The fourth consideration is that the current study was performed in the Netherlands. It should be noted that the conditions of receiving a disability claim, as well as subject characteristics, are different for different countries. However, self-employed mostly have a private insurance, which is usually not influenced by social insurance policies in countries. Finally, in the current study, persons were included in the study when their claims were already filed. The period before inclusion was up to 12 weeks, and because of this, the average duration of a claim was underestimated in the current study. This means that fewer people ended their disability claim after one year than stated in this article. However, taking these extra weeks into account in the analyses did not influence the effect of the prognostic factors that influenced claim duration (results not shown). On the other hand, by including only persons whose claims were already filed, we do not know the association of baseline factors and short-lasting claims.

## Conclusion

The prognostic factors associated with shorter claim duration in self-employed persons were personal factors (age over 40 years), physical body functions (having had no similar symptoms in the past, having long-lasting current symptoms of more than six months) and mental body functions (self-predicted return to work in more than one month or never, and job dissatisfaction). The few environmental prognostic factors we studied did not significantly influence the length of claim duration.

Although age and past symptoms were already known as prognostic factors for claim duration from other studies in self-employed, the duration of current symptoms, self-predicted return to work and job satisfaction were not yet known to influence claim duration in self-employed.

## Competing interests

The authors declare that they have no competing interests.

## Authors' contributions

JMR: was involved in the statistical analysis and drafted the manuscript. BMB: was involved in the conception and design of the study, coordinated the study, gave structural advice concerning the statistical analysis and helped to draft the manuscript. JH: was involved in the conception and design of the study, carried out the data collection and statistical analysis, and helped to draft the manuscript. EMMV: performed the data conformation, was involved in the statistical analysis and delivered critical improvements to the manuscript. JRA: was involved in the conception and design of the study, gave structural advice concerning the statistical analysis and delivered an important intellectual content regarding the revision of manuscripts. All authors read and approved the final manuscript.

## Pre-publication history

The pre-publication history for this paper can be accessed here:

http://www.biomedcentral.com/1471-2458/11/945/prepub
